# Evaluation of clear aligners as surgical splints in orthognathic surgery: A clinical study

**DOI:** 10.6026/973206300221850

**Published:** 2026-03-31

**Authors:** Sree Ram Subba Reddy Gudimetla, Rajshree Borah, Zeeshan Mubashir, Samir Mansuri, Salman Pasha, Durga Prasad K, Rahul Tiwari

**Affiliations:** 1 Department of Oral Maxillofacial surgery, Sree Mithra Dental and MaxFace Specialists, Tanuku andhra Pradesh, India; 2 Department of Dentistry, Tinsukia Medical College and Hospital, Makum, Assam, India; 3 Department of Orthodontics and Dentofacial Orthopaedics, Al Jouf Specialised Dental Center, Ministry of Health, Sakaka, Al Jouf Province, Kingdom of Saudi Arabia; 4 Department of Oral Maxillofacial surgery, Al Kuwait Hospital, Dubai, United Arab Emirates; 5 Department of Orthodontics and dentofacial orthopedics, AME'S Dental college, Bijangera road, Raichur, Karnataka, India; 6 Department of Oral and Maxillofacial Surgery, C.K.S. Theja Dental College, Renigunta Road, Tirupati, Andhra Pradesh, India; 7 Department of Oral and Maxillofacial Surgery, RKDF Dental College and Research Centre, Sarvepalli Radhakrishnan University, Bhopal, Madhya Pradesh, India

**Keywords:** Clear aligner, orthognathic surgical procedure, surgical stabilizer, virtual surgical planning (VSP), maximum precision

## Abstract

The digital orthodontic-surgical workflows are increasingly incorporating clear aligners. However, evidence supporting the use of aligner-based appliances as surgical splints for intraoperative use is insufficient. In this prospective study, patients who underwent bimaxillary orthodontic surgery were assigned to either surgical splints made of clear aligners (CASS) or traditional CAD/CAM printed occlusal surgical splints (CCS) as well as postoperative maxillary positioning accuracy was assessed. CASS showed a significant lower requirement to adjust intraoperatively and comparable maxillary positioning error as compared to CCS. The operational workflow metrics favor CASS without the loss of early occlusal precision. Clear aligners are able to be used as splints for surgery in certain orthognathic patients with the ability to predict intraoperative handling and accuracy in position.

## Background:

Use of VSP digitally printed splints for orthognathic surgery has led to enhanced pre-surgical predictability and efficiency, with several studies demonstrating clinically acceptable jaw positioning, along with workflow advantages when compared to conventional means [1]. Concurrently, clear aligner orthodontic regimens have evolved into perioperative orthognathic therapy such as segmental maxillary surgeries requiring good retention and occlusal control [2]. Emerging evidence indicates that clear aligners may have acceptable occlusal results in orthognathic surgery and promote reliable finishing if given to properly selected patients [3]. A randomized controlled trial also demonstrated both periodontal and quality of life benefits up to 1 year after surgery for patients following aligner therapy versus fixed appliances, showing further patient-centred advantages of removable systems [4, 5]. Therefore, it is of interest to report and evaluate the clinical applicability, intraoperative handling characteristics and early maxillary transfer accuracy of clear aligner-derived appliances when used as surgical splints in orthognathic surgery.

## Materials and Methods:

This prospective, two-arm clinical study was conducted in a tertiary maxillofacial surgery unit. Institutional clinical governance approval was obtained prior to study initiation and written informed consent was collected from all participants. Adults aged 18-35 years diagnosed with skeletal Class II or Class III dentofacial deformities and planned for Le Fort I (single-piece) with or without bilateral sagittal split osteotomy (BSSO) were included, provided virtual surgical planning (VSP) and complete preoperative digital records were available. Patients were excluded if they had syndromic conditions, cleft-related osteotomies, planned segmental Le Fort I procedures, distraction osteogenesis, or a history suggesting poor compliance with aligner therapy.

Participants were allocated to either a clear aligner-derived surgical splint (CASS) protocol, using aligners of 0.75-1.0 mm thickness reinforced with occlusal stops and trimmed to avoid interference with the surgical field, or a control group receiving conventional CAD/CAM 3D-printed occlusal splints fabricated from VSP (intermediate and/or final splints as required). The primary outcomes were intraoperative splint fit assessed by a surgeon-rated fit score (0-10) and the requirement for intraoperative adjustment (yes/no). Secondary outcomes were 1-week maxillary transfer accuracy (linear deviation, in mm, assessed at the incisal and bilateral molar points), the operative time cost due to splint handling (in min) and early postoperative occlusal discrepancy requiring elastics beyond normal protocol (yes/no). Continuous variables were presented as mean ± SD and compared between the two groups by independent t-test; categorical data was analyzed by chi-square test or Fisher exact test as appropriate, with p<0.05 considered statistically significant.

## Results:

A total of 44 patients were checked. No major perioperative complications were attributed to splint type. Baseline comparability was demonstrated between groups. Mean age was similar (24.6±3.8 vs 25.1±4.2 years; p=0.66), with comparable sex distribution (63.6% vs 59.1% female; p=0.76). Skeletal Class III deformity predominated in both arms (68.2% vs 63.6%; p=0.75). Most patients underwent bimaxillary surgery (81.8% versus 86.4%; p=0.68). Planned maxillary advancement was similar (3.4±1.6 vs 3.6±1.5 mm; p=0.71) Table 1 (see PDF). Intraoperative fit scores were high and not significantly different (8.8±0.9 vs 8.5±1.0; p=0.28). However, CASS required fewer intraoperative adjustments than CCS (9.1% vs 31.8%; p=0.048) and added less operative time attributable to splint handling (3.1±2.4 vs 7.0±4.6 minutes; p=0.001). Early maxillary transfer accuracy was comparable at 1 week (1.2±0.6 vs 1.3±0.6 mm; p=0.54), with similar rates of early occlusal discrepancy (13.6% vs 18.2%; p=0.68) Table 2 (see PDF).

## Discussion:

This study suggests that clear aligner-derived surgical splints can be clinically viable for intraoperative transfer in selected orthogenetic cases, with a meaningful reduction in adjustment burden and time cost while maintaining early maxillary positioning precision. These findings align with the broader observation that digital splint parameters especially manufacturing tolerances and offset strongly influence intraoperative seating and transfer fidelity; controlled evaluations show that moderate offset ranges may optimize precision for printed splints [6]. The emergence of new technologies, like AI-assisted or algorithmic design of splints also highlight that the design automation process can ensure an accurate fit and could improve intraoperative or chairside refinement [7]. Additionally, studies that evaluate the precision of virtually printed and designed intermediate splints show the idea that digital guides can achieve an acceptable level of clinically acceptable thresholds for maxillary repositioning with a technique-sensitive variation [8]. From a workflow perspective, our decreased "splint-related" time is clinically pertinent since operative efficacy is now viewed in conjunction with the accuracy of position. Reports on surgery first protocols that use clear aligners demonstrate the benefits of removable devices, but insist on the necessity of precise transfer instruments and stoppers for occlusal fixation [9]. The case-series research on using aligners for orthognathic surgery shows the effectiveness of aligners across a variety of deformities. It also highlights the necessity to standardize the trimming of aligners and reinforcement as well as indexing of the occlusion when aligners are utilized during perioperative procedures [10]. In more complex cases, like multi-segmental maxillary surgery have suggested aligner-supported strategies that are carefully supervised and controlled strategies, proving that the selection of indications and workflow control are crucial [11]. Modern navigational concepts that are integrated with splints made of CAD/CAM have demonstrated improvements in intraoperative maxillary control, suggesting that splints may function as an adjunctive verification device instead of a singular "transfer-only" device [12]. Randomized research that compares VSP against conventional planning has shown tangible accuracy advantages for digital paths for specific endpoints and constant improvements to digital transfer methods [13]. But evidence syntheses concluding that, even though aligners perform as well as fixed appliances in orthognathic paths, the quality and amount of direct evidence is still limited and an accurate interpretation is required [14, 15]. In sum, our findings suggest clear aligners to be surgical splints that can be used in appropriately selected bimaxillary patients; however larger multicenter studies that include more time to monitor stability and standard fabrication protocols are required.

## Conclusion:

Clear aligners can be used as splints for surgery in orthographic surgery, with a high intraoperative fit and fewer adjustments compared to conventional Splints that are CAD/CAM. The early maxillary transfer accuracy was comparable across the different methods in this clinical group. The next step is to standardize the design of aligner-splints and validate results in larger multicenter studies with long-term stability evaluation.

## Advancement to knowledge:

This prospective clinical study provides direct comparative evidence (2020-2025 contexts) that clear aligner-derived surgical splints demonstrate comparable early maxillary transfer accuracy to CAD/CAM printed splints, while significantly reducing intraoperative adjustment requirements and splint-related operative time, thereby supporting their feasibility as workflow-efficient transfer devices in selected orthognathic cases.
